# Novel inhibitor discovery and the conformational analysis of inhibitors of listeriolysin O via protein-ligand modeling

**DOI:** 10.1038/srep08864

**Published:** 2015-03-09

**Authors:** Jianfeng Wang, Xuan Zhou, Shui Liu, Gen Li, Bing Zhang, Xuming Deng, Xiaodi Niu

**Affiliations:** 1 Key Laboratory of Zoonosis, Ministry of Education, Department of Food Quality and Safety, College of Veterinary Medicine, Jilin University, Changchun, China

## Abstract

Increasing bacterial resistance to available antibiotics makes the discovery of novel efficacious antibacterial agents a priority. A previous report showed that listeriolysin O (LLO) is a critical virulence factor and suggested that it is a target for developing anti-virulence drugs against *Listeria monocytogenes* infections. In this study, we report the discovery of LLO natural compound inhibitors with differential activity by using hemolysis assay. The mechanism of action of the inhibitors was consistent with that of fisetin, a natural flavonoid without antimicrobial activity, which we showed in our previous report via molecular simulation. Furthermore, a substantial increase in anti-hemolytic activity was observed when the single bond (C1-C2) was replaced by a double bond (C1-C2) in the inhibitor molecule. This change was based on the decomposition of the ligand-residue interaction, which indicated that the double bond (C1-C2) in the inhibitors was required for their inhibition of LLO. The current MD simulation work provides insights into the mechanism by which the compounds inhibit LLO at the atomic level and will be useful for the development of new, selective LLO inhibitors.

L*isteria monocytogenes* (*L. monocytogenes*), the causative agent of listeriosis, is a gram-positive opportunistic pathogen that is mainly prevalent in the elderly, pregnant women, neonates and debilitated or immunocompromised patients, although it can also be present in normal individuals^1^. This bacterial pathogen is capable of crossing the intestinal, blood-brain and placental barriers after being ingested by the host, which can cause gastroenteritis, meningitis and abortions, with up to a 25–35% mortality rate^2^. The establishment of a robust infection is largely dependent on the production of virulence factors, such as internalin, listeriolysin, phospholipases and ActA protein, at the correct time and within the correct host environment. Following internalin-mediated entry into the host cells, the secreted protein, listeriolysin O (LLO), and phosphatidylinositol-specific phospholipase C (PI-PLC) facilitate the escape of *L. monocytogenes* from the vacuoles to the cytosol, where the bacteria replicate rapidly. Subsequently, the expression of ActA, a surface protein, enables the pathogen to exploit the host's actin cytoskeleton to power movement within and between cells, without exiting from the cells^3^. Among these stages, LLO-mediated escape of the pathogen from the vacuole into the cytosol of infected cell is indispensable for bacterial virulence^4^.

LLO, a member of the cholesterol-dependent cytolysin (CDC) family of membrane-penetrating toxins, is an essential virulence factor of *L. monocytogenes* because it can block phagosome-lysosome fusion by disrupting the membrane of the organelle, thus preventing the entrapped bacteria from being degraded in the phagolysosomal system^5^. However, in the cytosol, the concentration and activity of LLO is tightly regulated to prevent the killing of the infected cell, permitting the bacteria to evade extracellular immune system factors, such as the complement system and antibodies^6^. Although multifaceted activities are continuously being discovered^7^, the critical role of LLO for the pathogenicity of *L. monocytogenes* has been well characterized. Previous studies in which the inactivation of *hly* (the gene encoding LLO) was shown to significantly reduce the virulence of the pathogens^8^,^9^, the strain complemented with a plasmid carrying only *hly* displayed a hemolytic phenotype identical to that of the wild-type strain and was fully virulent^10^. According to Edelson et al., macrophages infected in the presence of an anti-LLO mAb that could neutralize LLO-mediated pore formation showed a significant reduction in intracellular *L. monocytogenes* growth and the passive administration of the antibody could provide resistance to *L. monocytogenes* infection in mice^11^,^12^. A previous study in our lab demonstrated that fisetin, a natural compound without anti-bacterial activity, inhibits the virulence of *L. monocytogenes* by attenuating the hemolytic activity of LLO^13^. Moreover, as LLO is not required for bacterial growth, targeting LLO would lead to milder evolutionary pressure for the development of resistance. Therefore, we hypothesized that screening natural compound inhibitors for inhibition of LLO could contribute to anti-virulence drug discovery by providing optimal host defense against *L. monocytogenes* infections.

In our previous study, we reported that the direct engagement of fisetin to the Loop2 and Loop 3 of LLO blocks the binding of cholesterol (CHO), an essential structural component of animal cell membranes that is required for the oligomerization of LLO, to LLO. This subsequently reduces the oligomerization of LLO, thus inhibiting its hemolytic activity. Here, we found that five natural compounds with similar structures, myricetin (Myr), morin (Mor), baicalein (Bac), chrysin (Chr) and naringenin (Nar), possess different inhibitory effects on the hemolytic activity of LLO. Computational biology assays and mutagenesis assays were employed to investigate the mechanism by which the inhibitors attenuated the hemolytic activity of LLO and the structure activity relationship of these natural compounds, which would benefit our understanding on drug discovery that targets LLO. The results from our study suggest that the double bond (C1-C2) is one of the key moieties in the inhibitors of LLO, and therefore, the compounds with the double bond (Myr, Mor and Bac) may be more promising candidate for the design of novel and potent inhibitors of LLO.

## Results

### Identifying LLO inhibitors

**Figure 1:**
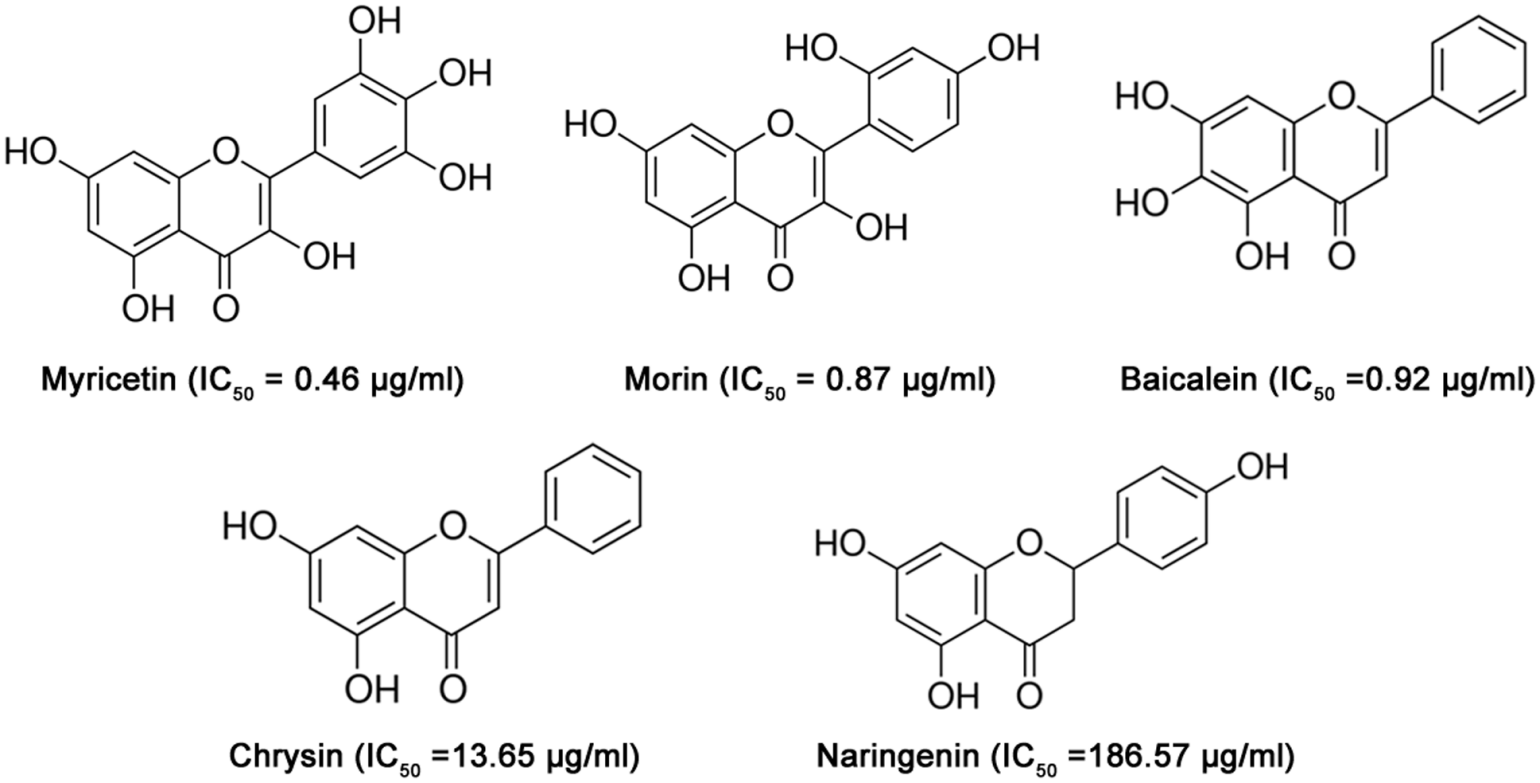
The chemical structures of the LLO inhibitors used for molecular simulation and binding free energy calculations.

**Figure 2:**
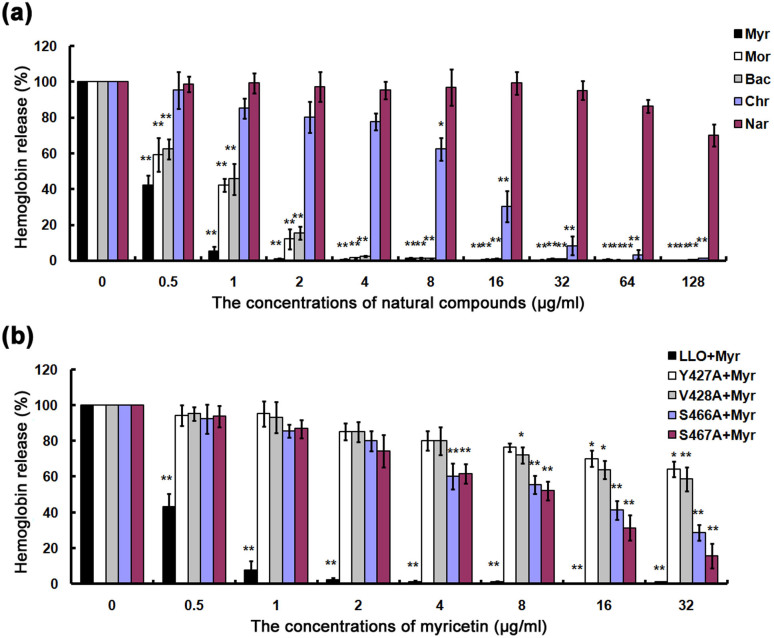
The inhibitory effect of five natural compounds on LLO activity. (a) Purified rLLO pretreated with the indicated concentrations of natural compounds was incubated with defibrinated sheep erythrocytes and hemolysis activity was determined by the release of hemoglobin at OD543. (b) Hemolysis assay was performed as described above to examine the inhibitory effects of myricetin on the hemolysis induced by LLO and its mutants. Bars represent the standard deviation (*P < 0.05, **P < 0.01; two tailed Student's t-test).

LLO has been proposed to be a promising target for the development of antilisteriosis drugs. Screening for inhibitors of LLO would facilitate the process of developing anti-LLO drugs for the treatment of infections. In the present study, we focused on five natural compounds, Myr, Mor, Bac, Chr and Nar (Fig. 1), identified in a screen of over 100 natural compounds for antagonism of the hemolytic activity of LLO. These five inhibitors belong to the flavonoid family and share structural similarities, but have with different inhibitory activities (Fig. 2a). Under our experimental conditions, the concentrations required for 50% inhibition (IC50) were 0.46, 0.87, 0.92, 13.65 and 186.57 μg/ml for Myr, Mor, Bac, Chr and Nar, respectively (Fig. 2a). Myr displayed the strongest inhibitory action among these five natural compounds (Fig. 2a).

### The interactions between the inhibitors and LLO predicted by three-dimensional modeling

The results from our functional analyses strongly suggested the direct targeting of LLO by five natural compound inhibitors. Next, we explored the mechanism of action of these compounds by studying their interactions with the toxin by molecular modeling. The process of the molecular modeling has been reported in previous papers^14^,^15^.

**Figure 3:**
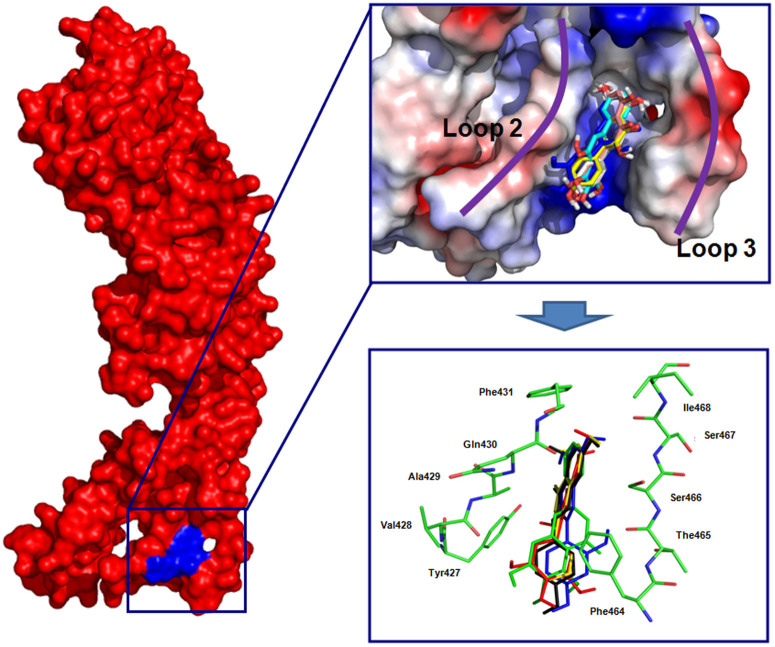
The interactions between inhibitors and LLO predicted by molecular modeling.

An overlay of the five modeling structures revealed that Myr, Mor, Bac, Chr and Nar bound LLO with the same binding mode (Fig. 3). Given the conservation of binding interactions between LLO and the shared components in Myr, Mor, Bac, Chr and Nar, we subsequently focused our discussion on the structure of the LLO-Myr complex, which has the highest activity. Information from the modeled structure of LLO-Myr derived from the docking results was used for the 200-ns molecular dynamics simulation, based on which the preferential mechanism of binding between Myr and LLO was established. These analyses revealed that hydrogen bonds and electrostatic interactions were involved in the formation of the LLO-Myr complex, in which two binding cavities, Loop 2 (L2) and the Loop 3 (L3) of the toxin, made direct contact with the Myr ligand (Fig. 3). Our modeling also indicated that the 4*H*-chromen-4-one moiety of the Myr could form strong interactions with the side chains of residues Tyr427, Val428, Gln430 and Phe431 in the L2 region. In addition, Myr is proximal to the residues, Phe464, Thr465, Ser466, Ser467 and Ile468 (L3 region), which suggests a strong stacking interaction between these residues and Myr. Due to the binding of inhibitors in the cavity of L2 and L3, the fluctuation of L2 was restricted, which led to an increase in the distance between L2 and L1. Such an increase made LLO less optimal for engaging CHO and this lower affinity for CHO consequently lowered its lytic activity. This mechanism is consistent with fisetin, a natural flavonoid without antimicrobial activity, which was identified in our previous report^13^.

**Figure 4:**
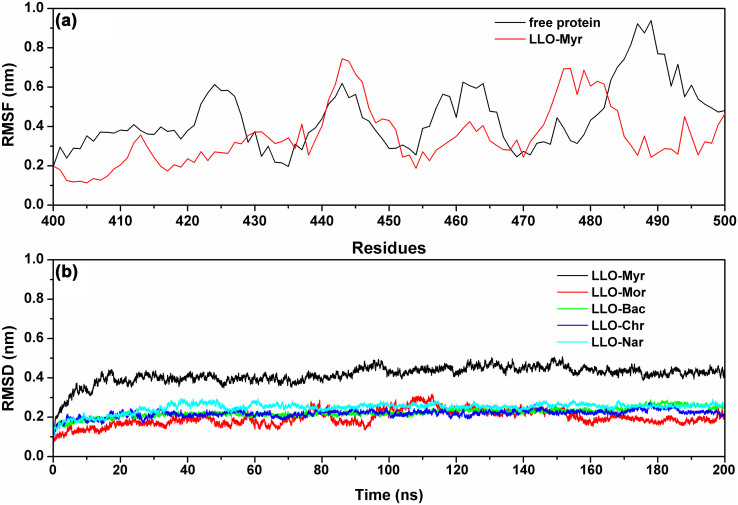
(a) RMSF of the residue positions over the last 50-ns simulations with respect to their initial position for the LLO protein in the free protein and LLO-Myr system. (b) The RMSDs displayed by the backbone atoms of the protein during MD simulations of LLO-Myr, LLO-Mor, LLO-Bac, LLO-Chr and LLO-Nar are presented.

During the last 100 ns of the simulation, the fluctuation patterns were different between the complexes and the free protein in the 420–470 region of LLO, which overlapped with the predicted binding sites (residues 420–470) (Fig. 4). Compared to the RMSF values calculated for free LLO, the engagement with Myr led to a lower flexibility of all the residues in the LLO binding site (with RMSF values less than 0.6 nm) (Fig. 4). Similar to Myr, in LLO-Mor, LLO-Bac, LLO-Nar and LLO-Chr complexes, the ligands bound to LLO in the same binding sites (Fig. 3). The only difference was that the distances between the 4*H*-chromen-4-one moiety and Tyr427 of LLO was longer than that of the LLO-Myr complex (1.30 nm, 1.50 nm, 1.75 nm and 1.85 nm for Mor, Bac, Chr and Nar, respectively). It is likely that the stacking interactions between the 4*H*-chromen-4-one moiety of inhibitors and Tyr427 (L3 region) are responsible for the potency and selectivity of Myr, Mor, Bac, Chr and Nar. This is evident from anti-hemolytic activity of inhibitors of inhibitors, which is consistent with the data from the modeling. Therefore, Mor, Bac and Chr were also identified as potent inhibitors of LLO. Taken together, our simulation revealed that residues 420–470 play important roles in the stabilization of the LLO binding cavity in the complexes with Myr, Mor, Bac Chr and Nar.

### Identification of the LLO residues important for interacting with inhibitors

To identify LLO residues important for the binding of inhibitors, we employed a combined approach that used the MM-GBSA calculation method and fluorescence spectroscopy quenching to predict the binding free energies of LLO-ligands^16^,^17^.

**Figure 5:**
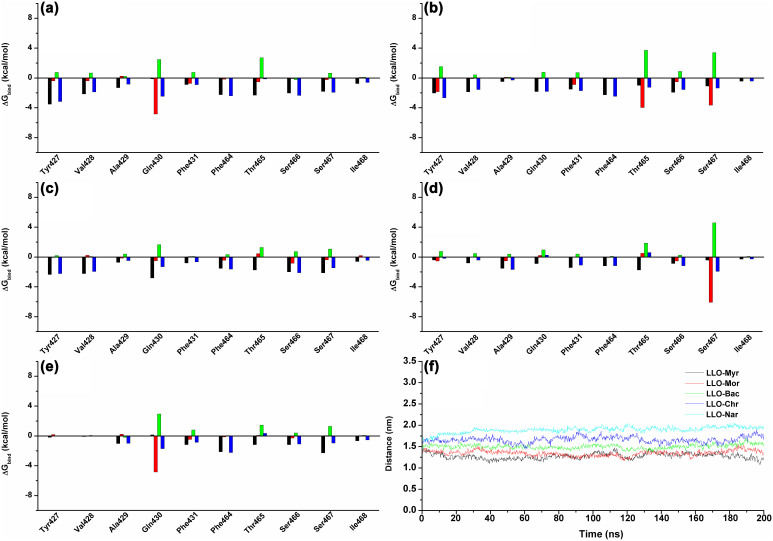
The decomposition of the binding energy on a per-residue basis in the binding sites of the LLO-Myr complex (a), LLO-Mor complex (b), LLO-Bac complex (c), LLO-Chr complex (d) and LLO-Nar complex (e). (black histograms: ΔE_vdw_; red histograms: ΔE_ele_; green histograms: ΔE_sol_; blue histograms: ΔE_total_). The distances between Tyr427 and the 4H-chromen-4-one moiety of ligands in the complexes are shown as a function of time (f).

Among these, Tyr427 and Val428 had appreciable van der Waal interactions of <**−**2.0 kcal/mol with Myr, Mor, Bac and Chr because of the close proximity between these residues and the benzene ring of inhibitors. An exception was Nar (**−**1.0 kcal/mol). It was indicated that L2 can anchor the right side of Myr, Mor, Bac and Chr (Fig. 5a–d) based on the stacking interactions. However, Phe464, Thr465, Ser466 and Ser467 appeared to have strong van der Waal interactions of ≤−2.5 kcal/mol with the five compounds, strongly suggesting that the L3 region of LLO can anchor Myr, Mor, Bac, Chr and Nar (Fig. 5a–d).

To validate these predictions, we first modeled the complexes formed with ligands and the Y427A, V428A, S466A and S467A mutants, and used the information as the preliminary structure for the MD simulations and trajectories analysis. Our results indicated that the four mutants bound to these five ligands in a manner similar to the wild type toxin. Predictably, the interactions between the ligands and the mutated residues were weaker than those observed in the wild type complexes.

The MM-GBSA calculation indicated that the four mutants bound to ligands with lower affinity than their wild type counterparts (Table 1). By fluorescence spectroscopy quenching^16^,^17^, we measured ΔG_bind_ and the number of binding sites between the ligands and the four mutants. These results were highly consistent with those obtained by computational methods, thus indicating the importance of these four residues in the interactions between LLO and the ligands (Table 2). We also examine the inhibitory effects of Myr, the compound had the highest LLO inhibitory activity, on LLO and its mutants. As expected, the mutants (Y427A, V428A, S466A and S467A) were less sensitive to Myr in the inhibition of hemolytic activity (Fig. 2b). These results indicate that the information generated by the MD simulation on the LLO-ligands complexes is reliable and that these two molecules likely engage in the manner predicted by the modeling.

### The analysis of the structure activity relationship of inhibitors

By performing the molecular dynamics (MD) simulations, free energy calculations and mutagenesis assays described previously^14^, we believe that the MD simulations generated reliable models for the LLO-inhibitors complexes. The predicted binding modes of ligands with LLO are illustrated in Fig. 3, and show the key residues around ligands (residues Tyr427, Val428, Gln430, Phe464, Ser466 and Ser467).

Next, the analysis of the ligand-residue interaction decompositions based on the Molecular Mechanics Generalized Born Surface Area (MM-GBSA) method indicated that Tyr427 had appreciable van der Waal interactions of ~**−**3.0 kcal/mol with Myr, Mor and Bac because of the close proximity between the residue and the 4*H*-chromen-4-one moiety of ligands (Fig. 3). However, in the LLO-Chr and LLO-Nar complexes, the interactions between Tyr427 and the 4*H*-chromen-4-one moiety of ligands were weaker. The decrease in the binding energy was due to the longer distance between Tyr427 and the 4*H*-chromen-4-one moiety of ligands. Next, the distances between Tyr427 and the 4*H*-chromen-4-one moiety of ligands in the complexes were calculated during the MD simulated trajectory, as shown in Fig. 5f. The average distances for LLO-Myr, LLO-Mor and LLO-Bac complexes were 1.25 nm, 1.30 nm and 1.50 nm, respectively. However, the average distance for LLO-Chr and LLO-Nar was 1.75 nm and 1.85 nm, respectively. Dynamic fluctuations in the distance between Tyr427 and the 4*H*-chromen-4-one moiety of ligands directly affected the values of the binding energy. The difference in distance between Tyr427 and the 4*H*-chromen-4-one moiety of ligands was due to whether the 4*H*-chromen-4-one moiety could be parallel to the benzene ring of Tyr427. In the LLO-Myr, LLO-Mor, LLO-Bac, LLO-Chr and LLO-Nar complexes, the 4*H*-chromen-4-one moiety of ligands were parallel to the benzene ring of Tyr427, and could form the strong π-π interactions. However, due to the lack of the double bond, C1-C2, the 2,3-dihydrochromen-4-one of Nar could not be parallel to the benzene ring of Tyr427 and thus, could not form the π-π interactions. Based on the above data, we believe that the double bond (C1-C2) is one of the key moieties in the inhibitors of LLO. This was confirmed by experimental data (Fig. 2a).

## Discussion

LLO (Listeriolysin O) is one of the emerging targets in the treatment of antibacterial infections and is mainly associated with multiple manifestations, such as bacteremia, meningitis and gastroenteritis in humans or animals^5^. In our previous study^13^, we reported that fisetin, a natural flavonoid without antimicrobial activity, could bind with LLO directly and inhibit its hemolytic activity by blocking its binding to cholesterol and reducing its oligomerization. In this study, we found that several natural compounds could inhibit the hemolytic activity of LLO more strongly. To identify the critical chemical features of natural compounds responsible for inhibiting LLO activity, MD simulations, site-specific mutagenesis and the fluorescence-quenching method were used to explore the ligand-protein binding sites. Furthermore, the MM-PBSA method was performed to determine the associated free energy profiles of such interaction.

Of all the small structural changes we had explored, the most important breakthrough discovery was a double bond (C1-C2) to single bond (C1-C2) replacement on the 4*H*-chromen-4-one moiety. As shown in Fig. 1 and Fig. 2a, the IC_50_ from Myr, Mor, Bac and Chr (IC_50_ = 0.46 μg/ml for Myr, IC_50_ = 0.87 μg/ml for Mor, IC_50_ = 0.92 μg/ml for Bac and IC_50_ = 13.65 μg/ml for Chr) represents a several-fold increase in potency from Nar (IC_50_ > 180 μg/ml). To analyze their structural properties, we compared the computational models of LLO-Myr, LLO-Mor, LLO-Bac, LLO-Chr and LLO-Nar in the binding cavity of LLO.

As expected, the inhibitors could bind to the loop2 region and loop3 region, indicating that the 4H-chromen-4-one moiety of the inhibitors could form strong interactions with the side chains of residues Tyr427, Val428 and Gln430 in the L2 region and that the pyrocatechol group of the inhibitors is proximal to residues Phe464, Ser466 and Ser467 (L3 region), which suggests strong interactions between these residues and inhibitors. Although all the ligands could bind to LLO, the details of the combinations between LLO and the inhibitors are different. For example, in Myr, Mor, Bac and Chr, the 4*H*-chromen-4-one moiety of the ligands are parallel to the benzene ring of Tyr427, and can form the strong π-π interactions, resulting in an increase in binding affinity. However, due to the lack of double bond (C1-C2), Nar showed the 2,3-dihydrochromen-4-one in a nonplanar orientation relative to the benzene ring of Tyr427, and did not form the π-π interactions. Based on the findings with the promising the natural flavonoids, Myr, Mor, Bac, Chr and Nar, we discovered that the bond (C1-C2) replacement from a single to double bond produced substantial improvements in anti LLO activity. Hence we suggest the use of Myr, Mor, Bac and Chr as tool compounds by medicinal chemists who are interested in working on LLO inhibitors.

In summary, based on computational approaches, five ligands were used to find the critical chemical features that can inhibit the activity of LLO. Based on the ligand-residue interaction decompositions analysis, a highly predictive model double bond (C1-C2) was selected as one of the key moieties. The inhibitors with the double bond on the 4H-chromen-4-one moiety had increased biochemical potency of 405-fold, 214-fold, 202-fold and 13-fold, compared to Nar, which has a single bond (C1-C2). Therefore, the compounds with the double bond on the 4H-chromen-4-one moiety may be the good candidates for the design of novel and potent inhibitors of LLO.

## Methods

### Preparation of recombinant LLO and its mutants

#### (i) Construction of plasmids encoding wild-type (WT)-LLO, Y427A-LLO, V428A-LLO, S466A-LLO and S467A-LLO

The truncated *hly* gene, encoding a mature protein of 26-529 amino-acid residues, was amplified from the genomic DNA of *L. monocytogenes* strain, EGD, which was kindly provided by Dr Masao Mitsuyama (Kyoto University Graduate School of Medicine, Japan). For insertion into the pET21a^+^ vector, the following two oligonucleotide primers, containing Nde I in sense and Xho I in antisense, were used in the PCR: sense, 5′-GCGCCATATGGATGCATCTGCATTCAATAAAG-3′ and anti-sense, 5′-GCGCCTCGAGTTCGATTGGATTATCTACTTTATTAC-3′. The PCR product was digested with the restriction endonucleases mentioned above, and ligated into the pET21a^+^ vector to generate pET21a^+^LLO.

The Y427A, V428A, S466A and S467A mutations were introduced using the QuikChange site-directed mutagenesis kit (Stratagene, CA, USA) on pET21a^+^LLO. The primers used to introduce the mutations include the following: Y427A, 5′-CACTCTGGAGGAGCGGTTGCTCAATTC-3′ (forward) and 5′- GAATTGAGCAACCGCTCCTCCAGAGTG-3′ (reverse); V428A, 5′-CTGGAGGATACGCGGCTCAATTCAAC-3′ (forward) and 5′- GTTGAATTGAGCCGCGTATCCTCCAG-3′ (reverse); S466A, 5′-CTAGCTCATTTCACAGCGTCCATCTATTTG-3′ (forward) and 5′- CAAATAGATGGACGCTGTGAAATGAGCTAG-3′ (reverse) and S467A, 5′-CATTTCACATCGGCGATCTATTTGCCAG-3′ (forward) and 5′- CTGGCAAATAGATCGCCGATGTGAAATG-3′ (reverse). The mutated codons are underlined. All mutations were verified by double strand DNA sequencing.

#### (ii) Expression and purification of WT-LLO and its mutants

The recombinant plasmids mentioned above were transformed into *Escherichia coli* strain BL21(DE3) (Novagen). When the cells were grown in LB broth plus ampicillin at 37°C to early log phase (OD600 = 0.7), IPTG was then added to a final concentration of 0.5 mM to induce the T7 RNA polymerase gene at 25°C for 20 hr. Then, the cells were pelleted, resuspended in in a lysis buffer (1 × PBS, 1 mM DTT and 1 mM PMSF) and lysed by sonication. Following 1 h of centrifugation at 12,000 rpm, the cell-free supernatant was loaded onto a self-packed His-affinity column (GE Amersham). The His6-tagged LLO was eluted from the column with 250 mM imidazole, and further purified on a Superdex 75 16/60 column (GE Healthcare) with storage buffer (35 mM Na3PO4, 125 mM NaCl, pH 5.5). The purity and identity of the proteins were analyzed by SDS-PAGE, immunoblot or by MALDI-TOF mass spectrometry. The proteins were stored at −80°C prior to subsequent applications.

### Hemolysis assay

A hemolysis assay was employed to assess the inhibitory effect of the tested natural compounds on the hemolytic activity of LLO. Briefly, 100 μl of purified LLO or its muatants, with a concentration of 100 ng/ml, was preincubated in microtiter plates in the presence of the natural compounds at 37°C for 15 min before adding 100 μl of defibrinated sheep erythrocytes (5 × 10^6^ cells/ml) to each well. The reaction mixture was further incubated at 37°C for 30 min, and then centrifuged (5,500 g, 4°C, 1 min) to remove the erythrocytes. The hemolytic activity was determined by measuring the OD543 of the cell-free supernatant. The samples treated with 1% Triton X-100 used as 100% lysis controls, and hemolysis was expressed as the ratio of the OD543 of each sample relative to the complete lysis controls.

### Starting structures for MD simulations

The structure of LLO was taken from the X-ray crystal structure in the Protein Data Bank with PDB codes of 4CDB^18^, and the free protein obtained from the PDB (4CDB) was firstly equilibrated by a 100 ns molecular simulation on the solute, which was further used for the molecular docking with ligands. The geometrics of Myr, Mor, Bac, Chr and Nar were optimized at the B3LYP/6-31G* level by the Gaussian 03 program.

### Molecular docking

The standard docking procedures for LLO with Myr, Mor, Bac, Chr and Nar were performed using AutoDock4^19^,^20^,^21^.

### MD simulations

The MD simulations were performed for free LLO and five complex systems (LLO-Myr, LLO-Mor, LLO-Bac, LLO-Chr and LLO-Nar) using the Gromacs 4.5.2 software package based on the Amber99sb force field, and TIP3P was used as water model^22^,^23^. The particle mesh Ewald algorithm was used to describe the electrostatic term, and the all bond lengths were constrained by the LINCS algorithm^24^. The desired initial temperature was 300 K and the initial velocity was obtained from a Maxwellian distribution. The first equilibration run at NPT condition was used to adjust the density of the system^25^ (*P_0_* = 1 bar, coupling time τ_p_ = 0.5 ps). The protein and non-protein systems were coupled separately to the temperature bath. The MD conditions of the free LLO are consistent with that of complexes. The antechamber programs and AM1-BCC partial atomic charges from the Amber10 software were used to estimate the ligands^25^ (Myr, Mor, Bac, Chr and Nar).

### Calculation of binding free energy

In this work, Molecular Mechanis/Poisson-Boltzman Surface Area (MM-PBSA) approach supplied with the Amber 10 package was performed to calculate the binding free energies between LLO and ligands. Subsequently, the interaction between inhibitors and each residue in the binding site of LLO was analyzed using the MM-PBSA decomposition process supplied in the MM-PBSA module in Amber 10. The binding interaction of each ligand-residue pair includes three terms: the Van der Waals contribution (*ΔE_vdw_*), the electrostatic contribution (*ΔE_ele_*) and the salvation contribution (*ΔE_sol_*).

### Binding affinity determination of inhibitors with WT-LLO and its mutants

The binding constants (KA) of inhibitors to the binding site of WT-LLO, Y427A-LLO, V428A-LLO, S466A-LLO and K433A-LLO were measured using the fluorescence-quenching method. Fluorescence spectrofluorimetry measurements were carried out using a Horiba Jobin-Yvon Fluorolog 3-221 spectrofluorometer (Horiba Jobin-Yvon, Edison, NJ). The measurements were acquired using a 280-nm excitation wavelength with a 5-nm band-pass and a 345-nm emission wavelength with a 10-nm band-pass. The details of the measurements were described previously^13^,^14^.

### Statistical analysis

Data were expressed as mean ± SD (standard deviation) from three independent experiments. The significance of hemolysis assay results was determined using the two-tailed Student's t test. Differences were considered statistically significant when *P* < 0.05.

## Author Contributions

X.D.N., X.M.D. and J.F.W. conceived and designed the experiments. J.F.W., X.Z., S.L. and G.L. performed the experiments. B.Z. contributed reagents/materials/analysis tools. X.D.N., X.M.D. and J.F.W. wrote the paper.

## Figures and Tables

**Table 1:** Calculated binding free energy (kcal/mol) of WT-inhibitors, Y427A-inhibitors, V428A-inhibitors, S466A-inhibitors and S467A-inhibitors systems

	WT-LLO	Y427A	V428A	S466A	S467A
**Myr**	−11.4 ± 2.1	−6.8 ± 1.1	−7.1 ± 1.4	−7.5 ± 1.6	−7.9 ± 1.6
**Mor**	−10.7 ± 1.6	−6.4 ± 1.0	−7.0 ± 1.1	−7.6 ± 1.3	−6.5 ± 1.1
**Bac**	−9.4 ± 1.0	−5.9 ± 0.9	−6.2 ± 1.1	−6.9 ± 1.7	−5.8 ± 0.9
**Chr**	−8.8 ± 0.9	−5.6 ± 0.7	−5.4 ± 1.0	−6.4 ± 1.2	−6.0 ± 0.8
**Nar**	−7.7 ± 0.8	−5.5 ± 1.1	−4.7 ± 0.7	−5.7 ± 1.0	−4.4 ± 0.6

**Table 2:** The binding constants (K_A_) (1 × 10^5^) L·mol^−1^ of the LLO-inhibitors systems based on the fluorescence-quenching method

	WT-LLO	Y427A	V428A	S466A	S467A
**Myr**	4.21	1.97	2.05	2.14	2.34
**Mor**	3.98	2.04	1.98	1.91	2.01
**Bac**	3.84	1.89	1.88	1.84	1.86
**Chr**	3.59	1.81	1.90	1.77	1.84
**Nar**	3.14	1.74	1.84	1.69	1.71
